# A Survey on Evolutionary Algorithm Based Hybrid Intelligence in Bioinformatics

**DOI:** 10.1155/2014/362738

**Published:** 2014-03-06

**Authors:** Shan Li, Liying Kang, Xing-Ming Zhao

**Affiliations:** ^1^Department of Mathematics, Shanghai University, Shanghai 200444, China; ^2^Department of Computer Science, School of Electronics and Information Engineering, Tongji University, Shanghai 201804, China

## Abstract

With the rapid advance in genomics, proteomics, metabolomics, and other types of omics technologies during the past decades, a tremendous amount of data related to molecular biology has been produced. It is becoming a big challenge for the bioinformatists to analyze and interpret these data with conventional intelligent techniques, for example, support vector machines. Recently, the hybrid intelligent methods, which integrate several standard intelligent approaches, are becoming more and more popular due to their robustness and efficiency. Specifically, the hybrid intelligent approaches based on evolutionary algorithms (EAs) are widely used in various fields due to the efficiency and robustness of EAs. In this review, we give an introduction about the applications of hybrid intelligent methods, in particular those based on evolutionary algorithm, in bioinformatics. In particular, we focus on their applications to three common problems that arise in bioinformatics, that is, feature selection, parameter estimation, and reconstruction of biological networks.

## 1. Introduction

During the past decade, large amounts of biological data have been generated thanks to the development of high-throughput technologies. For example, 1,010,482 samples were profiled and deposited in Gene Expression Omnibus (GEO) database [1] by the writing of this paper, where around thousands of genes on average were measured for each sample. The recently released pilot data from the 1000 genomes project indicate that there are 38 million SNPs (single-nucleotide polymorphism) and 1.4 million biallelic indels within the 14 populations investigated [2]. Beyond that, other large-scale omics data, for example, RNA sequencing and proteomics data, can be found in public databases and are being generated everyday around the world. Despite the invaluable knowledge hidden in the data, unfortunately, the analysis and interpretation of these data lag far behind data generation.

It has been a long history that intelligent methods from artificial intelligence were widely used in bioinformatics, where these approaches were utilized to analyze and interpret the big datasets that cannot be handled by biologists. For example, in their pioneering work, Golub et al. utilized self-organizing maps (SOMs) to discriminate acute myeloid leukemia (AML) from acute lymphoblastic leukemia (ALL) based only on gene expression profiles without any prior knowledge [3]. Later, support vector machine was employed to classify 14 tumor types based on microarray gene expression data [4]. Except for diagnosis, intelligent methods have been exploited to identify biomarkers [5], annotate gene functions [6], predict drug targets [7, 8], and reverse engineering signaling pathways [9], among others.

Despite the success achieved by standard intelligent methods, it is becoming evident that it is intractable to analyze the large-scale omics data with only single standard intelligent approaches. For example, when diagnosing cancers based on gene expression profiles, low accuracy is expected if a traditional classifier, for example, linear discriminant analysis (LDA), is employed to classify the samples based on all the genes measured. This phenomenon is caused due to the “large *p* small *n*” paradigm which arises in microarray data, where there are generally around 20 thousand of genes or variables that were measured for each sample while only tens or at most hundreds of samples were considered in each experiment. In other words, there are very few samples while a much larger number of variables are to be learned by the intelligent methods, that is, the curse of dimensionality problem. Therefore, it is necessary to employ other intelligent techniques to select a small number of informative features first, based on which a classifier can be constructed to achieve the desired prediction accuracy. Such hybrid intelligent methods, that is, the combination of several traditional intelligent approaches, are being proved useful in analyzing the big complex biological data and are therefore becoming more and more popular.

In this paper, we survey the applications of hybrid intelligent methods in bioinformatics, which can help the researchers from both fields to understand each other and boost their future collaborations. In particular, we focus on the hybrid methods based on evolutionary algorithm due to its popularity in bioinformatics. We introduce the applications of hybrid intelligent methods to three common problems that arise in bioinformatics, that is, feature selection, parameter estimation, and molecular network/pathway reconstruction.

## 2. Evolutionary Algorithm

In this section, we first briefly introduced evolutionary algorithm, which is actually a family of algorithms inspired by the evolutionary principles in nature. In the evolutionary algorithm family, there are various variants, such as genetic algorithm (GA) [10, 11], genetic programming (GP) [12], evolutionary strategies (ES) [13], evolutionary programming (EP) [14], and differential evolution (DE) [15]. However, the principle underlying all these algorithms is the same that tries to find the optimal solutions by the operations of reproduction, mutation, recombination, and natural selection on a population of candidate solutions. In the following parts, we will take genetic algorithm (GA) as an example to introduce the evolutionary algorithm.

**Figure 1:**
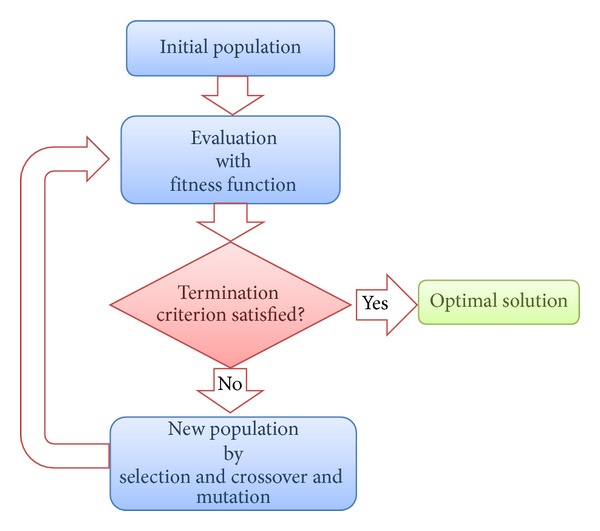
The schematic flowchart of genetic algorithm.

Figure 1 presents a schematic flowchart of genetic algorithm. In genetic algorithm, each candidate solution should be represented in an appropriate way that can be handled by the algorithm. For example, given a pool of candidate solutions *X* of size *M*, *X* = {**x**_1_,**x**_2_,…,**x**_*M*_}^*T*^, a candidate solution **x**_*i*_, that is, an individual, can be represented as a binary string **x**_*i*_ = [0,0, 1,0,…, 1]. Take feature selection as an example; each individual represents a set of features to be selected, where element 1 in the individual means that the corresponding feature is selected and vice versa. After the representation of individuals is determined, a pool of initial solutions is generally randomly generated first.

To evaluate each individual in the candidate solution pool, a fitness function or evaluation function *F* is defined in the algorithm. The fitness function is generally defined by taking into account the domain knowledge and the optimal objective function to be solved. For instance, the prediction accuracy or classification error can be used as fitness function. If an individual leads to better fitness, it is a better solution and vice versa.

Once the fitness function is determined, the current population will go through two steps: selection and crossover and mutation. In selection step, a subset of individual solutions will be selected generally based on certain probability, and the selected solutions will be used as parents to breed next generation. In the next step, a pair of parent solutions will be picked from the selected parents to generate a new solution with crossover operation; meanwhile, mutation(s) can be optionally applied to certain element(s) within a parent individual to generate a new one. The procedure of crossover and/or mutation continues until a new population of solutions of similar size is generated.

The genetic algorithm repeats the above procedure until certain criterion is met; that is, the preset optimal fitness is found or a fixed number of generations are reached. Despite the common principles underlying the evolutionary algorithm family, other variants of the algorithm may have implementation procedures that are different from the genetic algorithm. For example, in differential evolution, the individuals are selected based on greedy criterion to make sure that all individuals in the new generation are better than or at least as good as the corresponding ones in current population. Another alternative of the traditional genetic algorithm, namely, memetic algorithm (MA), utilizes a local search technique to improve the fitness of each individual and reduce the risk of premature convergence.

Since the evolutionary algorithm starts with a set of random candidate solutions and evaluates multiple individuals at the same time, the risk of getting stuck in a local optimum is reduced. Furthermore, the evolutionary algorithm can generally find optimal solutions within reasonable time, thereby becoming a popular technique in various fields.

## 3. Feature Selection in Bioinformatics

**Figure 2:**
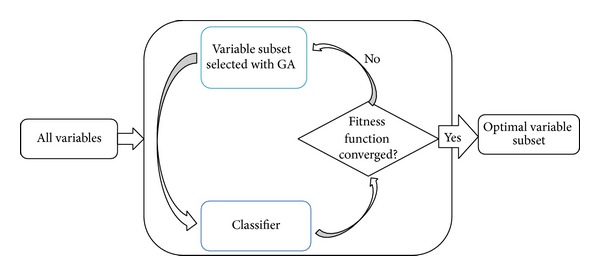
The flowchart of feature selection based on GA and classifier.

In bioinformatics, various problems are equivalent to feature selection problem. For example, in bioinformatics, biomarker discovery is one important and popular topic that tries to identify certain markers, for example, genes or mutations, which can be used for disease diagnosis. It is obvious that biomarker identification is equivalent to feature selection if we consider genes or mutations of interest as variables, where the informative genes or mutations are generally picked to discriminate disease samples from normal ones. However, it is not an easy task to select a few informative variables (generally <20) from thousands or even tens of thousands of features. Under the circumstances, the evolutionary algorithm has been widely adopted for identifying biomarkers along with other intelligent methods.  Figure 2 depicts the procedure of feature selection with GA, where GA generally works together with a classifier as a wrapper method and the classifier is used to evaluate the selected features in each iteration. For example, Li et al. [16] utilized genetic algorithm and *k*-nearest neighbor (KNN) classifier to find discriminative genes that can separate tumors from normal samples based on gene expression data, and robust results were obtained by the hybrid GA/KNN method. Later, Jirapech-Umpai and Aitken [17] applied the GA/KNN approach to leukemia and NCI60 datasets, where the prediction results by the hybrid method are found to be consistent with clinical knowledge, indicating the effectiveness of the hybrid method. Since the simple genetic algorithm (SGA) often converges to a point in the search space, Goldberg and Holland adopted the speciated genetic algorithm, which controls the selection step by handling its fitness with the niching pressure, for gene selection along with artificial neural network (SGANN) [18]. Benchmark results show that SGANN reduces much more features than SGA and performs pretty well [19]. Recently, the hybrid approaches that, respectively, combined Pearson's correlation coefficient (CC) and Relief-F measures with GA were proposed by Chang et al. [20] to select the key features in oral cancer prognosis. These hybrid approaches outperform other popular techniques, such as adaptive neurofuzzy inference system (ANFIS), artificial neural network (ANN), and support vector machine (SVM). In addition to gene selection, the hybrid methods involving evolutionary algorithm have been successfully used to identify SNPs associated with diseases [21, 22] and peptides related to diseases from proteomic profiles [23–25].

Beyond biomarker identification, the evolutionary algorithm based hybrid intelligent methods have also been successfully applied to other feature selection problems in bioinformatics. For example, Zhao et al. [26] proposed a novel hybrid method based on GA and support vector machine (SVM) to select informative features from motif content and protein composition for protein classification, where the principal component analysis (PCA) was further used to reduce the dimensionality while GA was utilized to select a subset of features as well as optimize the regularization parameters of SVM at the same time. Results on benchmark datasets show that the hybrid method is really effective and robust. The hybrid method that integrates SVM and GA was also successfully used to select SNPs [27] and genes [28] associated with certain phenotypes and predict protein subnuclear localizations based on physicochemical composition features [29]. Recently, the hybrid SVM/GA approach was also utilized for selecting the optimum combinations of specific histone epigenetic marks to predict enhancers [30]. Saeys et al. predicted splice sites from nucleotide acid sequence by utilizing the hybrid method combining SVM and estimation of distribution algorithms (EDA) that is similar to GA [31]. Nemati et al. further combined GA and ant colony optimization (ACO) together for feature selection, and the hybrid method was found to outperform either GA or ACO alone when predicting protein functions [32]. In addition, Kamath et al. [33] proposed a feature generation with an evolutionary algorithm (FG-EA) approach, which employs a standard GP algorithm to explore the space of potentially useful features of sequence data. The features obtained from FG-EA enable the SVM classifier to get higher precision.

Feature selection is an important topic in bioinformatics and is involved in the analysis of various kinds of data. The hybrid methods that utilize the evolutionary algorithm have been proven useful for feature selection when handling the complex biological data due to their efficiency and robustness.

## 4. Parameter Estimation in Modeling Biological Systems

In bioinformatics, one biological system can be modeled as a set of ordinary differential equations (ODEs) so that the dynamics of the systems can be investigated and simulated. For example, Zhan and Yeung modeled a molecular pathway with the following ODEs [34]:

(1) $$\begin{array}{c} \dot{x}\left(t\right)=f\left(x\left(t\right),u\left(t\right),\theta \right),\\ x\left(t_{0}\right)=x_{0},\\ y\left(t\right)=g\left(x\left(t\right)\right)+\eta \left(t\right), \end{array}$$

where *x* ∈ *R* ^*n*^ is the state vector of the system, *θ* ∈ *R* ^*k*^ is a parameter vector, *u*(*t*) ∈ *R* ^*p*^ is the system's input, *y* ∈ *R* ^*m*^ is the measured data, *η*(*t*) ~ *N*(0, *σ* ^2^) is the Gaussian white noise, and *x*_0_ denotes the initial state. *f* is designed as a set of nonlinear transition functions to represent the dynamical properties of the biological system and *g* is a measurement function. It can be seen that, to make the model work, it is necessary to estimate the parameters in the model, which can be transformed into an optimization problem as follows:

(2) $$\begin{array}{c} P:\underset{\hat{\theta },{\hat{x}}_{0}}{\min}\overset{N-1}{\underset{j=0\;}{\sum }}\;\overset{n}{\underset{i=1}{\sum }}w_{ij}{||y_{i}\left(t_{j}\right)-{\hat{y}}_{i}\left(t_{j}\;|\;\hat{\theta }\right)||}_{l}, \end{array}$$

where $\hat{y}\left(t_{j}\right)=g\left(\hat{x}\left(t_{j}|\hat{\theta }\right)\right)$, ||·||_*l*_ denotes the *l*-norm, $\hat{x}\left(t_{j}|\hat{\theta }\right)$ is the variable at time *t*_*j*_ with parameter $\hat{\theta }$, *w*_*ij*_ denotes the weight, and $\hat{y}$ means the estimated value. The problem *P* could be solved easily by employing the evolutionary algorithms [35–37]. For example, Katsuragi et al. [38] employed GA to estimate the parameters required by the simulation of dynamics of the metabolite concentrations, and Ueda et al. [39] applied the real-coded genetic algorithm to find the optimal values of the parameters. Recently, in order to improve the accuracy of parameter estimation, Abdullah et al. [40] proposed a novel approach that combines differential evolution (DE) with the firefly algorithm (FA), which outperformed other well-known approaches, such as particle swarm optimization (PSO) and Nelder-Mead algorithm.

In biological experiments, most data observed are measured at discrete time points while the traditional ODE model is a set of continuous equations, which makes it difficult to estimate the parameters in an accurate way. Therefore, the S-system, which is a type of power-law formalism and a particular type of ODE model, was widely used instead. For example, Savageau and Rosen [41] modeled the genetic network with the following S-system model:

(3) $$\begin{array}{c} \frac{dX_{i}}{dt}=\alpha_{i}\overset{n+m}{\underset{j=1}{\prod }}X_{j}^{g_{ij}}-\beta_{i}\overset{n+m}{\underset{j=1}{\prod }}X_{j}^{h_{ij}}, \end{array}$$

where *X*_*i*_ denotes the variable or reactant, *n* and *m*, respectively, denote the number of dependent and independent variables, *α*_*i*_ and *β*_*i*_ are nonnegative rate constants, and *g*_*ij*_ and *h*_*ij*_ are kinetic orders. Here, the parameters *α*_*i*_, *β*_*i*_, *g*_*ij*_, and *h*_*ij*_ must be estimated. To optimize the parameters, Tominaga and Okamoto [42] utilized GA to approach the optimization problem with the following evaluation function *E*:

(4) $$\begin{array}{c} E=\overset{n+m\;}{\underset{i=1\;}{\sum }}\overset{T}{\underset{t=1}{\sum }}{\left(\frac{X_{i}^{\prime }\left(t\right)-X_{i}\left(t\right)}{X_{i}\left(t\right)}\right)}^{2}, \end{array}$$

where *T* is the number of sampling points and *X*_*i*_(*t*) and *X*_*i*_′(*t*), respectively, denote experimentally observed and estimated value at time *t* for *X*_*i*_. Later, Kikuchi et al. [43] found that it is difficult to estimate all the parameters from limited time-course data of metabolite concentrations. Hence, they changed the evaluation function *E* as follows:

(5) E=∑i=1 n+m ∑t=1T(Xi′(t)−Xi(t)Xi(t))2 +c(n+m)T{∑i,j|gij|+∑i,j,i≠j|hij|},

where *c* is a penalty constant that balances the two evaluation terms. Moreover, they adopted the simplex operations [44] instead of the random ones to accelerate the searching in GA. Considering only a few genes affecting both the synthesis and degradation processes of specific genes, Noman and Iba [45] further simplified the evaluation function as follows:

(6) $$\begin{array}{c} E_{i}=\overset{T}{\underset{t=1}{\sum }}{\left(\frac{X_{i}^{\prime }\left(t\right)-X_{i}\left(t\right)}{X_{i}\left(t\right)}\right)}^{2}+c\overset{n+m-1}{\underset{j=1}{\sum }}\left(\left|K_{i,j}\right|\right), \end{array}$$

where *K*_*i*,*j*_ is the kinetic order of gene *i*. With this objective function, they adopted a novel hybrid evolutionary algorithm, namely, memetic algorithm (MA) [46], that combines global optimization and local search together to find the optimal solutions. Considering that the traditional S-system can only describe instantaneous interactions, Chowdhury et al. [47] introduced the time-delay parameters to represent the system dynamics and refined the evaluation function as follows:

(7) $$\begin{array}{c} E=\overset{T}{\underset{t=1}{\sum }}{\left(\frac{X_{i}^{\text{cal}}\left(t\right)-X_{i}^{\exp}\left(t\right)}{X_{i}^{\exp}\left(t\right)}\right)}^{2}+B_{i}\times C_{i}\frac{2N}{2N-r_{i}}, \end{array}$$

where *r*_*i*_ is the number of all actual regulators, *B*_*i*_ is a balancing factor between the two terms, and *C*_*i*_ is the penalty factor for gene *i*. The trigonometric differential evolution (TDE) technique was adopted to estimate the set of parameters because of its better performance than other traditional evolutionary algorithms.

Parameter estimation is a key step in mathematical modeling of biological systems, which is however a nontrivial task considering the possible huge search space. Due to its excellent searching capability, the evolutionary algorithm is able to help determine the model parameters along with other intelligent approaches.

## 5. Molecular Network/Pathway Reconstruction

Recently, the network biology that represents a biological system as a molecular network or graph is attracting more and more attention. In the molecular network, the nodes denote the molecules, for example, proteins and metabolites, while edges denote the interactions/regulations or other functional links between nodes. Although it is easy to observe the activity of thousands of molecules at the same time with high-throughput screening, it is not possible to detect the potential interactions/regulations between molecules right now.

Under the circumstances, a lot of intelligent methods have been presented to reconstruct the molecular networks, such as Boolean network and Bayesian network. When reconstructing the molecular networks, one critical step is to determine the topology of the network to be modeled, based on which the interactions/regulations between molecules can be investigated. The topology determination problem can be treated as an optimization problem that is ready to be solved with the help of the evolutionary algorithm.

**Figure 3:**
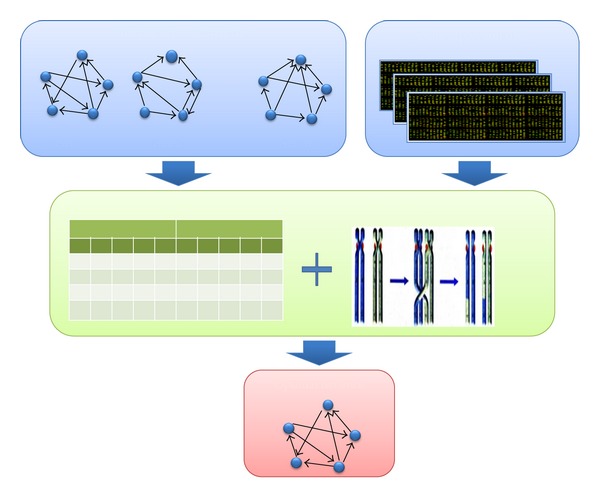
The reconstruction of gene regulatory network based on gene expression with the hybrid method consisting of Boolean network and evolutionary algorithm.

Take a gene regulatory network as an example; Figure 3 shows the flowchart of reconstructing the regulatory network based on gene expression data by utilizing Boolean network and evolutionary algorithm. In the example, we want to reconstruct the regulatory circuit that controls the gene expression of five genes. Since at least one edge exists while at most 10 edges exist in the network, the number of possible network structures will be *M* = ∑_*i*=1_^10^ *C*_10_^*i*^ = 2^10^ − 1 ≈ 2^10^. It is impossible to validate all network topologies by biologists in lab. With appropriate fitness function, the evolutionary algorithm is able to identify the optimal network structure that fits best the gene expression data, where the consistence between network topology and gene expression data is evaluated with Boolean network based on certain rules.

Repsilber et al. [48] modeled the gene regulatory network with a Boolean model as a directed acyclic graph *G* = (*V*, *F*), where *V* = {*x*_1_, *x*_2_,…, *x*_*n*_} denotes the set of genes in the regulatory network and *F* = {*f*_1_, *f*_2_,…, *f*_*n*_} denotes the Boolean rules that describe the regulations between nodes (or genes). To determine the topology of the regulatory network that better fits the observed data, they employed GA with the following fitness function *f*:

(8) $$\begin{array}{c} f=\frac{1}{1+\left(1/D\right)\sum_{ijk}\delta_{ijk}^{2}}, \end{array}$$

where *δ*_*ijk*_ = (sim_data_*ijk*_ − network_output_*ijk*_) is the difference between the observed data and those estimated from the generated network. In this way, they successfully reconstructed the gene regulatory network that generates the expression profiles consistent with experiments.

Later, Mendoza and Bazzan [49] presented inconsistency ratio (IR) to evaluate each individual node in the network, where the IR is defined as follows:

(9) $$\begin{array}{c} \text{I}{\text{R}}_{i}=w^{-1}\overset{2^{K}}{\underset{k=1}{\sum }}\min\left(w_{k}\left(0\right),w_{k}\left(1\right)\right). \end{array}$$

Here, *k* = 1,2, 3,…, 2^*K*^ is the number of possible input combinations for a node, *w*_*k*_(0) denotes the weight of measurements with output of 0 while *w*_*k*_(1) denotes those with output of 1, and *w* is the sum of all weights. With the IR defined above, an evaluation function defined below was used to investigate the inconsistency between the network generated and the experimental data:

(10) $$\begin{array}{c} \phi =\frac{1}{1+\left(\sum_{i=1}^{N}{\text{IR}}_{i}/\left(N\times 0.5\right)\right)+\left(\text{NP}/N^{2}\right)}, \end{array}$$

where *N* × 0.5 denotes the maximum inconsistency to be generated by the network while (NP/*N* ^2^) is a penalty factor. With this evaluation function, the differential evolution (DE) approach was used to identify the optimal network structure [50].

Recently, to understand the signaling in distinct physiological situations, Terfve et al. [51] proposed a CellNOptR approach, which derives a Boolean logic model from a “prior knowledge network” and uses GA to search the optimal network structure that is consistent with the perturbation data. Later, Crespo et al. [52] employed Boolean logic model and genetic algorithm to predict missing gene expression values from experimental data and obtained promising results.

Although the Boolean network is simple and capable of handling large networks, it fails to provide quantitative information about regulations between molecules, which is however the key to understand the regulation process. In this case, the Bayesian network is widely adopted. Considering the expensive computation time required by Bayesian network, the evolutionary algorithm is widely used to determine the structures of the molecular networks modeled. In the Bayesian network, the molecular network is regarded as a directed acyclic graph described as follows:

(11) $$\begin{array}{c} P\left(x_{1},x_{2},\ldots ,x_{n}\right)=\overset{n}{\underset{i=1}{\prod }}P\left(x_{i}\;|\;\pi_{i}\right), \end{array}$$

where *x*_*i*_ denotes node *i* in the set of variables, that is, the molecules considered, and *π*_*i*_ denotes the parent node of *x*_*i*_. For example, Yu et al. [53] utilized GA to determine the optimal network structure consistent with experimental data along with the dynamic Bayesian network by defining an evaluation function based on Bayesian dirichlet equivalence (BDe) score and Bayesian information criterion (BIC) score. Later, Xing and Wu [54] employed the maximum likelihood (ML) score and the minimal description length (MDL) score as fitness values and determined the topology of gene regulatory networks with GA, where the regulatory network is modeled with Bayesian network. Recently, Li and Ngom [55] proposed a new high-order dynamic Bayesian network (HO-DBN) learning approach to identify genetic regulatory networks from gene expression time-series data and obtained the optimal structure of the networks with GA. In their method, the optimal structure $\hat{S}$ was estimated by the maximum likelihood as follows:

(12) $$\begin{array}{c} \hat{S}=\overset{}{\underset{\theta_{s}}{\int }}P\left(X\;|\;\theta_{s}\right)P\left(\theta_{s}\;|\;S\right)d\theta_{s}, \end{array}$$

where *X* = {*x*_1_, *x*_2_,…, *x*_*n*_} and *θ*_*s*_ = {*θ*_1_, *θ*_2_,…, *θ*_*n*_} is the parameter set.

In addition to Boolean and Bayesian networks, the Petri net [56] is also widely employed to reconstruct biological networks. For example, in the Petri net model of metabolic networks, the nodes named places denote metabolites or products while transitions representing reactions are edges, where the values accompanying transitions denote rate constants. The input places for a transition denote the reaction's reactants while the output places denote its products, and the value of a place can be represented by its corresponding amount of substance. If a transition is deleted, a reaction happens, in which reactants are consumed and products are yielded. To find the optimal solutions, Nummela and Juistrom [57] defined a fitness function *F* as follows:

(13) $$\begin{array}{c} F=\sum \frac{\left|c_{mi}-c_{mi0}\right|}{n_{m}n_{p}}+0.1\times n_{r}, \end{array}$$

where *c*_*mi*_ means the computed concentration of the *m*th metabolite at time *i*, *c*_*mi*0_ is the corresponding target concentration, *n*_*m*_ means the number of metabolites, *n*_*p*_ is the number of time steps, and *n*_*r*_ is the number of reactions. With the hybrid method combining the Petri net and GA, they successfully identified a network that is consistent with the simulated data. Later, Koh et al. [58] have also successfully employed this hybrid method to model the AKt and MAPK signaling pathways.

The molecular networks enable one to investigate the biological systems from a systematic perspective, whereas the network topology is the key to construct and understand the network. Accumulating evidence demonstrates that the hybrid heuristic methods involving evolutionary algorithm are able to help determine the network topology consistent with experimental data in an accurate way due to its significant efficiency.

## 6. Conclusions

In this paper, we surveyed the applications of hybrid intelligent methods, which combine several traditional intelligent approaches together, in bioinformatics. Especially, we introduced the hybrid methods involving evolutionary algorithm and their applications in three common problems in bioinformatics, that is, feature selection, parameter estimation, and reconstruction of biological networks. The evolutionary algorithm was selected here due to its capability of finding global optimal solutions and its robustness. The hybrid intelligent approaches that combine evolutionary algorithm together with other standard intelligent approaches have been proved extremely useful in the above three topics. We hope this review can help the researchers from both bioinformatics and informatics to understand each other and boost their future collaborations. We believe that, with more effective hybrid intelligent methods introduced in the future, it will become relatively easier to analyze the ever-growing complex biological data.
